# Growth curve mixed nonlinear models in quails

**DOI:** 10.1371/journal.pone.0287056

**Published:** 2023-06-09

**Authors:** Raimundo Nonato Colares Camargo Júnior, Cláudio Vieira de Araújo, Flávio Luiz de Menezes, Simone Inoe de Araújo, Naiana Leticia Pavan, Mérik Rocha-Silva, Welligton Conceição da Silva, José Ribamar Felipe Marques, André Guimarães Maciel e Silva, Hipócrates de Menezes Chalkidis, José de Brito Lourenço Júnior

**Affiliations:** 1 Institute of Veterinary Medicine, Postgraduate Program in Animal Science (PPGCAN), Federal University of Para (UFPA), UFRA, Brazilian Agricultural Research Corporation (EMBRAPA), Castanhal, PA, Brazil; 2 Federal University of Mato Grosso (UFMT), Mato Grosso, MT, Brazil; 3 State University of Piauí (UESPI), Floriano, PI, Brazil; 4 Brazilian Agricultural Research Corporation (EMBRAPA), Belém, PA, Brazil; 5 Amazon University Center, Santarém, Pará, Brazil; Ain Shams University Faculty of Agriculture, EGYPT

## Abstract

Our aim was to evaluate the use and application of different nonlinear mixed models, as well as to compare them with approach in nonlinear fixed models, for describing the growth curve of meat-type quails according to gender. A total of 15,002 and 15,408 records of males and females were used, respectively. The body weights were regressed on age of the animals using nonlinear models (Brody; Gompertz; Logistic, Morgan-Mercer-Flodin, Richards and Von Bertalanffy). All model parameters were considered fixed, whereas parameters related to asymptotic weight and maturity rate were fitted as random effects. The Bayesian Information Criterion was used to find the model of best fit. For both genders, the model that used the Morgan-Mercer-Flodin function with the inclusion of asymptotic weight as a random effect was considered the best-fitting model because it reduced the residual variance and increased the accuracy. Based on the lower absolute growth rate and growth velocity of male quails compared to that of females, it can be inferred that males should be slaughtered later. Given the results of this study, it can contribute to the current knowledge about animal yield, specifically at the best moment to slaughter and, this sense, improv the quality genetic of the populations in time.

## Introduction

The quail (*Coturnix coturnix*) has unique and pleasant meat and egg flavor characteristics [1–4], fast reproduction and fast return on invested capital [5–7]. For these reasons, quails were bred all over the world to produce meat, eggs or for both purposes, in European countries, in the East and in other Asian countries, respectively [8–12].

In Brazil, the creation of quails to produce meat and eggs has also become economically viable and for this reason has been developing more and more, enshrining Brazil as the fifth largest producer of quail meat in 2011 and the second largest producer of eggs [13–17], justifying why the number of birds and egg production has almost doubled in recent years [18–22].

The use of mathematical models in animal production allowed researchers to describe and understand the biological processes and to evaluate the response variable effects [23–29].

In this way, the nonlinear regression models showed themselves as indicated and more efficient in describing important biological phenomena in livestock, including those associated with the evaluation of the individuals’ body weights as a function of age. This fact allows the comparison of different animals in similar physiological states for identifying individuals with higher body weight at earlier ages; moreover, it is also possible to obtain the variance among individuals included in genetic evaluation programs [30–33].

One of the advantages of using nonlinear models is that they allow summarizing information of a data set of longitudinal nature from animals that are developing in different classes of fixed effects (diets, sex, environments) in a few numbers of parameters associated with a specific function that describes the trajectory of phenotypic development [34–37].

In this sense, studies involving meat-type quails in comparisons between models in different sex or diets have been used with the functions of Brody, von Bertalanffy, Logistics and Gompertz, of Richards and hyperbolic models [38–41] and also in broiler chickens [42–44]. As well as in the description to analyze the effect of environmental factors on the characteristics of the egg production curve in broiler breeders using the compartmental function [45–47].

Nonlinear mixed models have been used previously in studies involving quail growth curves [48–50]. In this context, with the use of these models is expected to obtain more precise estimate of parameters associated with animal growth.

The application of nonlinear mixed models for studying growth curves has provided a better fit for individual and mean growth curves compared with nonlinear fixed models[51–53]. It occurs because mixed models incorporate nonlinear functions, combining fixed and random effects, which allows for considering the variability among and within individuals. Therefore, it improves the accuracy of estimates that describe animal growth and provides a better interpretation of the factors affecting it, resulting in more accurate identification of the most productive individuals [54].

Pinho et al. [55], Giacomini Sari et al. [56] stated that mixed effect models incorporate one or more random-effect parameters in the model, which in most cases result in increased variance and correlation. It improves the goodness of model data fit, making mixed models more appropriate. The aim was to evaluate the use and application of nonlinear mixed models for describing the growth curve of meat-type quails, as well as, to describe the characteristics of the growth of the quails according to the gender, raised from Diamantina city, state of Minas Gerais, Brazil.

## Materials and methods

The experiment was approved by the Research Ethics Committee of the Centro Universitário da Amazônia (UNAMA), protocol n° 0003-87/2023 (CEUA).

A total of 30,410 body weight records from 15,002 male and 15,408 female meat-type quails (*Coturnix coturnix*) of the LF1 strain were used. The records were derived from the Quail Breeding Program of the Federal University of Jequitinhonha and Mucuri Valleys, from Diamantina city, state of Minas Gerais, Brazil.

The quails were raised on boxes with concrete floors with covered by wood shavings and were subsequently moved to cages. Quails were fed a diet consisting of 25% of crude protein and 2,900 kcal of ME kg-1 from hatching to 21 days; from 21 to 42 days old, they received a diet containing 24% CP and 2,925 kcal of ME kg-1. The birds were weighed weekly from hatching to slaughter (1, 7, 14, 21, 28, 35, and 42 days).

The body weight records (in grams) were regressed on age (in days) using nonlinear models (Brody; Gompertz; Logistic, Morgan-Mercer-Flodin, Richards and Von Bertalanffy). All nonlinear model parameters were considered fixed, whereas A e K parameters were fitted as random effects (Table 1).

**Table 1:** Fixed and mixed nonlinear models for the functions of Brody, Gompertz, Logistic, Morgan-Mercer-Flodin, Richars and Von Bertalanffy.

	Nonlinear model	Mixed A Random	Mixed K random
Brody	Y_t_ = A (1 –Be^-kt^)	(A+a_1_) (1 –Be^-kt^)	A (1 –Be^-(k+k1)t^)
Gompertz	Y_t_ = ${Ae}^{{-e}^{-B(kt)}}$	${(A+a_{1})e}^{{-e}^{-B(kt)}}$	${A+e}^{{-e}^{-B((k+k1)t)}}$
Logistic	Y_t_ = A (1 + Be^-kt^)^-1^	(A+a_1_) (1 + Be^-kt^)^-1^	A+ (1 + Be^-(k+k1)t^)^-1^
Morgan-Mercer-Flodin	Y_t_ = (AB+k t^m^)/(B+ t^m^)	((A+a_1_)B+k t^m^)/(B+ t^m^)	(A+B+(k+k_1_) t^m^)/(B+ t^m^)
Richars and Von Bertalanffy	Y_t_ = A (1 –Be^-kt^)^m^	(A+a_1_) (1 –Be^-kt^)^m^	A+ (1 –Be^-(k+k1)t^)^m^
	Y_t_ = A (1 –Be^-kt^)^3^	(A+a_1_) (1 –Be^-kt^)^3^	A+ (1 –Be^-(k+k1)t^)^3^

Yt body weight (in grams) at age “t”; "A" asymptotic weight or average weight at maturity; “B” constant of integration; "K" maturity rate and constant "m".

### Statistical analysis

The Bayesian Information Criterion was used to find the model of the best fit: BIC = -2log⁡(L)+p log⁡〖(n)〗, where log⁡(L) is the logarithm of the likelihood function, "n" is the sample size and "p" is the number of parameters in the model.

The following parameters were calculated based on the nonlinear model of best fit for describing the growth curve in quails, according to gender: 1) Absolute Growth Rate (AGR), obtained from the first derivative of the fitted nonlinear function; 2) Relative Growth Rate (RGR), obtained as the ratio between the AGR and the body weight predicted by the nonlinear function, expressed as the increase in body weight per day in relation to the predicted weight; and 3) Growth Velocity Rate (GV), defined as the variation in the rate of body weight gain over time (in days), obtained from the second derivative of the fitted nonlinear function.

All analyses were performed by the Statistical Analysis System—SAS [57] package by using the Nlmixed procedure (Table 2).

**Table 2:** Number of observations (N) means and standard deviations (SD) for body weight of male and female quails at different ages.

Age (days)	Male (g)			Female (g)		
	N	Mean	SD	N	Mean	SD
1	2702	9.16	0.99	2782	9.22	1.06
7	2463	28.14	6.41	2486	28.57	6.57
14	2451	66.61	17.05	2504	67.66	18.08
21	2455	113.47	27.85	2526	115.91	30.53
28	2482	164.11	32.25	2569	169.36	35.23
35	2449	211.51	30.35	2541	219.58	33.10
42	1522	238.16	28.46	1555	246.05	32.71

## Results

Overall, for both genders, it is shown that the best fitting models according to the Bayesian Information Criterion (BIC) where those that included the asymptotic weight parameter as a random effect (Morgan-Mercer-Flodin (MMF); Gompertz and Von Ber-talanffy (Table 3), in that order).

**Table 3:** Estimation of parameters obtained in the models that used the functions: Morgan-Mercer-Flodin, Gompertz and Von Bertalanffy, considering asymptotic weight as random at age “t”.

Gender	Models	BIC
	Mixed model of the Morgan-Mercer-Flodin function	
Males	y_t_ = (((593,00+17,81)(0,0011))+12,55t^-1,717^))/(0,0011+ t^-1,717^)	122637
Females	y_t_ = ((623,00+18,59)0,001+14,29t^-1,74^)/(0,001+ t^-1,74^)	127181
	Mixed model of the Gompertz function	
Males	$y_{t}=(361,61+2,773)e^{\left({-e}^{3,548(0,0054t)}\right)}$	122695
Females	$y_{t}=(390,39+3,118)e^{\left({-e}^{3,353(0,0052t)}\right)}$	127381
	Mixed model of the Von Bertalanfy function	
Males	y_t_ = (518,04+6,83) (1–0,746e^-0,03t^)^3^	122858
Females	y_t_ = (577,31+8,15) (1–0,745e^-0,029t^)^3^	127588

**Fig 1:**
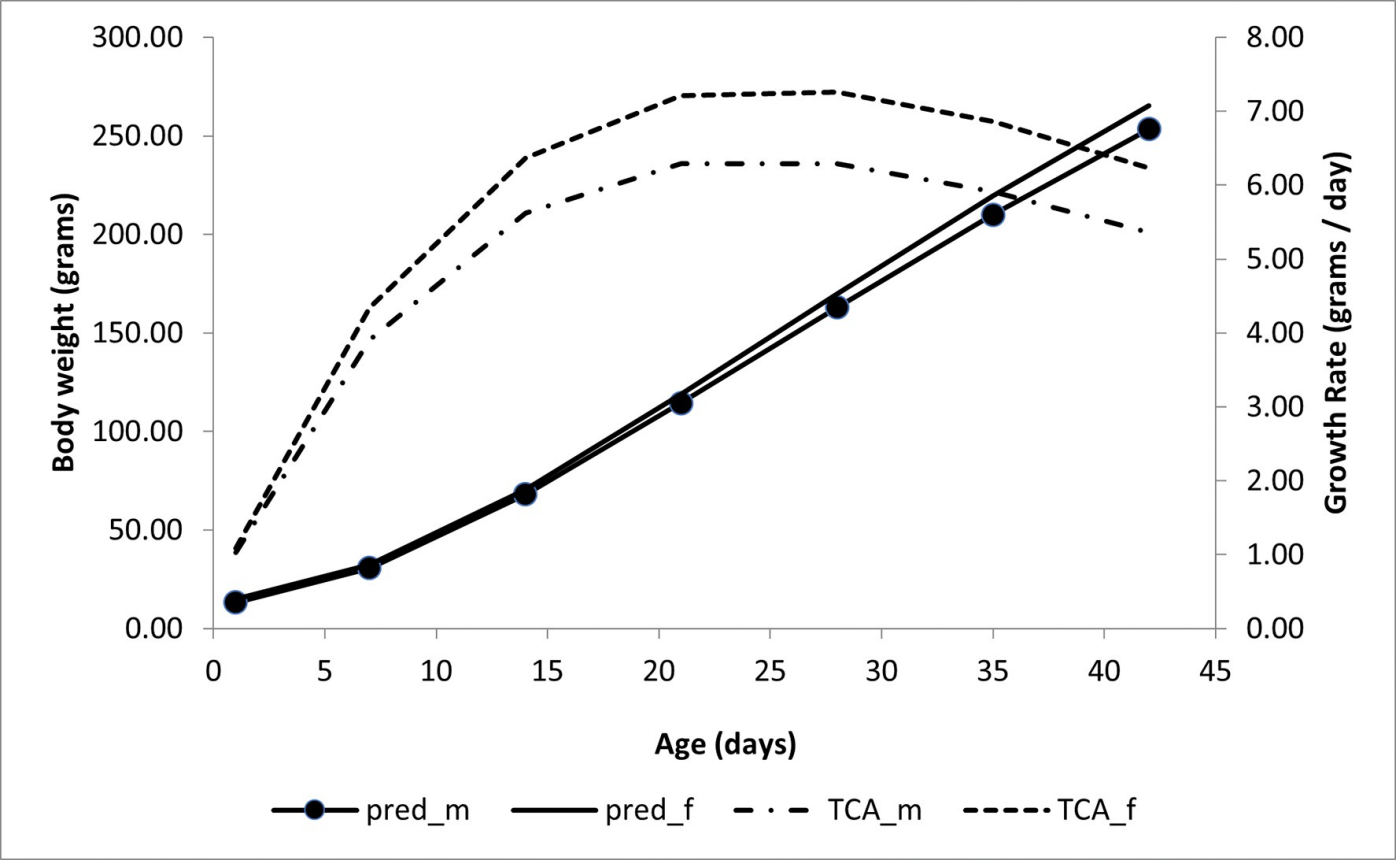
Predicted body weight and absolute growth rate (AGR) as a function of age (days) using the Morgan-Mercer-Flodin model for male and female quails, with asymptotic weight parameter being random. **Note:** pred_m, and pred_f are the body weights of males and females, respectively. TCA_m, and TCA_f are the absolute growth rates of males and females, respectively.

When exploring the model using the Morgan-Mercer-Flodin function with the asymptotic weight parameter as a random effect, the inflection point (IP = ((B(m-1))/(m+1))^(1/m)) was reached at 24 days of age for males and at 25 days of age for females (Fig 1). In this situation, the body weight of 135 and 148 grams corresponded to 22.8% and 23.6% of the asymptotic weight, for males and females, respectively.

The quails showed the maximum absolute growth rate at the inflection point of 6.3 and 7.3 grams/day, for males and females, respectively.

Similarly, using the Gompertz function in mixed models by comparison between of female quail of the lineage meat and the posture, Santos et al. (2018) showed that the meat line presented a higher growth rate (6.95 g/day) than the lineage for laying (3.65 g/day). The relative growth rate was higher for lineage for laying (0.15%) in relation to the lineage for meat (0.13%).

The Relative Growth Rate (RGR) in males decreased over time, but without being less than zero. At the inflection point, the quails were weighing on average 134 grams and gaining approximately 6.9 grams per day; it represents a relative growth of 4.95% of their daily weight at the inflection point (Fig 1).

## Discussion

The flaws in the process of convergence in nonlinear mixed models were also verified by Galeano-Vasco et al. [58], van der Klein et al. [59], Aggrey [60] when studying the Brody and Von Bertalanffy models with random effects for the growth curve in commercial laying hens.

Both fixed and mixed Brody models showed flaws in the process of convergence for both genders. The same behavior was reported by Mota et al. [61], Lupi et al. [62], Ghavi Hossein-Zadeh [63], Mohammadi et al. [64], which verified limitations of the Brody function when used to model data from commercially exploited animals, mainly due to their great growth rate.

In studies conducted by Wang and Zuidhof [65], Beiki et al. [66], Zuidhof [67], Afrouziyeh et al. [68], the best fit for modelling the growth curve of broiler chickens was obtained using the mixed Gompertz growth model. According Gürcan and Kaplan [69], the MMF is the second-best model among the four best models according to BIC values.

The mixed nonlinear model permits consideration of the heterogeneity among individuals arising from variables not measured through the inclusion of random effects in the model [49,70,71].

In all models, when considering parameter A as random, there was a reduction in the estimate of this parameter. That is because there is major variability between the individual values for this parameter, when considering a variance component associated with it, the variance is obtained between a minor amplitude, changing the average value of the parameter.

In this sense, the inclusion of parameter A as a random effect increased the accuracy of models by reducing the residual variance. In the stochastic modeling approach with the inclusion of random effects in nonlinear models, modeling the (co)variance structure of random parameters separates the variation that would be directed to the residual in a fixed model approach. In this case, the residual variance in the MMF fixed model is 19.79 grams for males, while the same estimate in the mixed model is equal to 11.54 grams (58% reduction). In the same sense, females showed estimates of 11.90 and 21.18 grams in mixed and fixed models, respectively (56% reduction).

Similarly, to this study, Karaman et al. [72] observed a reduction in the error variance of up to 72% by using mixed models instead of conventional nonlinear models and applying the logistic model to study the growth of meat-type quails.

In the model using the Morgan-Mercer-Flodin function, the highest values observed for predicted body weight and growth rate of females at the inflection point are unusual to species with sexual dimorphism, in which females reach higher body weight at maturity, with estimated asymptotic weights equal to 623.00 and 593.68 grams in females and males, respectively.

The absolute growth rate indicates the extent to which animal growth is accentuated relative to its lifespan. Therefore, it is necessary to explore this characteristic by ensuring full access of quails to feed, as well as avoiding stressful routines during this period of higher growth rate.

The relative growth rate (RGR) is a reflex of the absolute growth rate and represents the body weight gain per unit of time (in this case, grams per day) relative to body weight at a given point.

Overall, the observed RGR values indicated satisfactory growth rates. Therefore, this information can be used to monitor the development and overall state of quails. A group of animals with weight gains lower than 4% of the body weight tends to be affected by factors unfavorable to their development, such as poultry diseases.

It is observed that before the inflection point (at 24 days of age), the Growth Velocity (GV) is high and positive. After this point, there is a gradual deceleration in growth, where the relative growth (RG) maintains a nonlinear reduction until it approaches zero.

Quails at more advanced ages have lower relative growth, although it is greater than zero. Then, the animals are still growing even after 42 days, but at a lower velocity.

At the inflection point (25 days of age), females had an average daily gain of 7.8 grams with a mean body weight of 144 grams. Their relative growth rate represents 7.2 grams/day per body weight (gram); therefore, for each gram of body weight on a given day, 0.07 grams are being gained.

Females had a RGR greater than 10% from the third to the twelfth day of life. It was the period of highest RGR due to the accelerated weight gain associated with a lower body weight, approaching zero at the age of asymptotic weight.

Silva et al. [73], Pinheiro et al. [74], Wilkanowska and Kokoszyński [75] suggest that females should be slaughtered before males, which should be slaughtered at 42 days. Based on the relative growth rate of quails, our results corroborate that suggested by the authors, since the growth of females is more intense near the inflection point, with reduced development at the final stage.

When comparing the AGR of quails, it is observed that male quails were more de-veloped in the first days. At the fifth day of life, male and female quails had similar AGR (3.54 grams), and after that moment females showed greater growth velocity mainly dur-ing the third week of life, in parallel with the development of the reproductive system. In the last week of life (35 to 42 days), the mean growth velocity in females and males reached -0.07966 and -0.07904, respectively. It shows that females have an inflection point with a sudden, more intense acceleration, which reflects a sharp deceleration, with less regularity compared to males.

According to Krishnan [76], Abou-Kassem et al. [77], Retes et al. [78], quail females are heavier on average than males, with evident differences in size after the third week of life. The mean body weight of quails, regardless of gender, was similar until the 14 days of age. After that moment, a superiority in the body weight of females in relation to that of males was observed.

## Conclusions

The function of Morgan-Mercer-Flodin in no liner model with the inclusion of as-ymptotic weight as a random effect is the most accurate for describing the body develop-ment of meat-type quails as a function of age in both genders.

The lower maturity rate, absolute growth rate, and lower growth velocity show that males have slower development than females and, consequently, should be slaughtered later.
